# The Impact of Metabolic Dysfunction-Associated Steatotic Liver Disease on Autoimmune Hepatitis Outcomes: A Nationwide Analysis of 2,880 Records

**DOI:** 10.14309/ctg.0000000000000912

**Published:** 2025-09-12

**Authors:** Yassine Kilani, Mohammad Aldiabat, Kym Yves T. Sirilan, Ahmad Basil Nasir, Mahmoud Y. Madi, Wing-Kin Syn

**Affiliations:** 1 Division of General Internal Medicine, Department of Medicine, Saint Louis University School of Medicine, St. Louis, Missouri, USA;; 2 Department of Medicine, Washington University in St. Louis, St. Louis, Missouri, USA;; 3 Department of Medicine, New York Health + Hospitals | Lincoln - Weill Cornell Medical College affiliate, New York, New York, USA;; 4 Division of Gastroenterology & Hepatology, Department of Medicine, Saint Louis University School of Medicine, St. Louis, Missouri, USA;; 5 Department of Physiology, Faculty of Medicine and Nursing, University of Basque Country UPV/EHU, Vizcaya, Spain.

**Keywords:** autoimmune hepatitis, metabolic dysfunction-associated steatotic liver disease, metabolic syndrome

## Abstract

**INTRODUCTION:**

Despite the growing recognition of autoimmune hepatitis (AIH)—metabolic dysfunction-associated steatotic liver disease (MASLD) overlap, studies today are limited by small sample sizes. The aim of this study was to investigate the impact of MASLD on the outcomes of patients with AIH using large-scale real world data.

**METHODS:**

This cohort study used the TriNetX research network to identify US adults (≥18 years) with AIH. Patients were stratified into those with MASLD (AIH-MASLD cohort) and controls (AIH without MASLD). Propensity score matching (1:1) between AIH-MASLD and controls accounted for demographics, comorbidities, and treatments. Outcomes were classified as short-term (within 1 year after diagnosis) or long-term (within 10 years) outcomes.

**RESULTS:**

Among 4,798 records with AIH, 1,440 AIH-MASLD patients were propensity matched with 1,440 controls. AIH-MASLD patients demonstrated reduced 1-year risks of all-cause mortality (hazard ratio [HR] 0.66, 95% confidence interval [CI] 0.44–0.98) and immunosuppressive medication use (HR 0.69, 95% CI 0.63–0.76), along with increased 10-year risks of cirrhosis (HR 1.22, 95% CI 1.06–1.40) and hepatocellular carcinoma (HR 2.03, 95% CI 1.09–3.78) compared with controls.

**DISCUSSION:**

In summary, our study using real-world evidence showed a significant association between MASLD and worse clinical outcomes in patients with AIH. Future efforts should be targeted toward facilitating early detection and management of MASLD in patients with AIH.

## INTRODUCTION

Autoimmune hepatitis (AIH) is characterized by chronic autoimmune inflammation of the liver associated with the presence of circulating autoantibodies (e.g., antinuclear antibodies, smooth muscle antibodies), hypergammaglobulinemia (e.g., elevated immunoglobulin G levels), and interface hepatitis on liver histology (1–5). The global prevalence of AIH is approximately 10–25 cases per 100,000 individuals (6–10), and its incidence is rising worldwide (5). Several factors have been implicated in the loss of immune tolerance to self-antigens, including genetic (e.g., HLA-DR3 and HLA-DR4 alleles) and environmental factors such as drug exposure or viral infections (e.g., measles, herpes simplex viruses, Epstein-Barr viruses, hepatitis viruses, human immunodeficiency virus) (11,12). Untreated, progressive inflammation in AIH may lead to liver fibrosis and potentially cirrhosis (1).

By contrast, metabolic dysfunction-associated steatotic liver disease (MASLD), formerly known as nonalcoholic fatty liver disease, is the most common chronic liver disease (CLD) worldwide, affecting 25% of the general population (13), with the highest prevalence recorded in the Middle East (32%), South America (31%), and Asia (27%). MASLD is characterized by hepatic lipid accumulation because of increased free fatty acid intake, de novo lipogenesis, and impaired lipid export, collectively leading to lipotoxicity, oxidative stress, and inflammation (14). Manifestations range from simple hepatic steatosis to inflammation (e.g., metabolic dysfunction-associated steatohepatitis [MASH]), fibrosis, and potentially cirrhosis. There is a strong association between MASLD and metabolic syndrome, with prevalence up to 75% recorded in patients with metabolic syndrome (13). Owing to its typically asymptomatic clinical course, MASLD is commonly diagnosed incidentally through the detection of elevated liver enzymes or imaging findings of hepatic steatosis (15).

In this context of MASLD “pandemic,” studies estimate that the prevalence of MASLD among individuals with AIH is similar to that of the general population (13,16–21). However, significant diagnostic challenges in cases of AIH-MASLD overlap arise because of clinical, serological, and histological similarities between the 2 conditions (11). In fact, 12%–48% of individuals with MASLD were found positive for serum autoantibodies (11,22,23), potentially leading to false AIH diagnoses. Furthermore, MASH can present histologically with features suggestive with autoimmune diseases (e.g., AIH or primary biliary cirrhosis [PBC]), including interface hepatitis, steatosis, bile duct damage, and portal inflammation (11). In addition, standard therapeutic regimens for AIH primarily involve immunosuppression with glucocorticoids and azathioprine; however, these agents may exacerbate MASLD by increasing insulin resistance and promoting hepatic steatosis (16). Consequently, alternative immunosuppressive strategies, including mycophenolate mofetil, have been investigated (15). Additional focus on lifestyle interventions such as weight loss and exercise in AIH-MASLD could further limit disease progression.

However, despite the growing recognition of AIH-MASLD overlap, current studies are still limited by small sample sizes (11,15–18,24), warranting the need for large-scale studies of patients with AIH-MASLD overlap as compared with those without MASLD. Furthermore, current literature lacks a comprehensive assessment of the outcomes in patients with MASLD compared with those without. Therefore, the aim of this study was to investigate the impact of MASLD on the outcomes of patients with AIH using real-world data from a comprehensive national database.

## METHODS

### Data source

This retrospective cohort study used the TriNetX Analytics Network Platform (Cambridge, MA), a comprehensive global federated research network encompassing deidentified electronic health records across 126 million patients across 71 US healthcare organizations (HCOs) (25). This platform facilitates cohort selection and propensity score matching (PSM), allowing for comparative analysis (25). Rigorous quality assurance process is enforced during electronic health record extraction, ensuring standardized formatting before database inclusion. This study is therefore exempt from Institutional Review Board approval, as it involved publicly available deidentified data (25,26).

### Study population and variables

**Figure 1.**
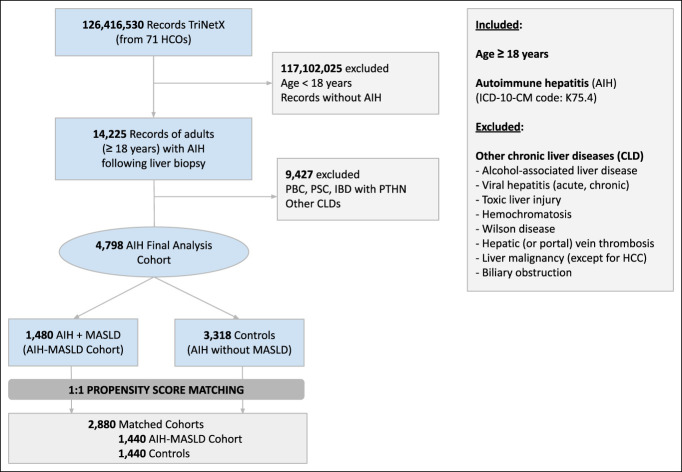
Flowchart of our study population. Matching was performed based on demographics (age, sex, and race/ethnicity), comorbid conditions (celiac disease), and treatment (prednisone, budesonide, azathioprine, 6-mercaptopurine, mycophenolate mofetil, tacrolimus, sirolimus, and cyclosporine). AIH, autoimmune hepatitis; CLD, chronic liver disease; MASLD, metabolic dysfunction-associated liver disease.

A real-time search and analysis of the US Collaborative Network in the TriNetX platform were conducted through July 30, 2025. Using* International Classification of Diseases, Ninth or Tenth Edition*, and *Clinical Modification/Procedure Coding System* (*ICD-9*/*10-CM*, *ICD-10-PCS*), along with Current Procedural Terminology (CPT) codes, we identified records of adults (≥18 years) who underwent a liver biopsy (CPT: 47000, 47100, 47001; *ICD-10-PCS*: 0FB00ZX, 0FB03ZX, 0FB04ZX, 0FB20ZX, 0FB23ZX, 0FB10ZX, 0FB13ZX, 0FB24ZX, 0FB14ZX) and were subsequently diagnosed with AIH (*ICD-9-CM*: 571.42 or *ICD-10-CM*: K75.4) (Supplementary Table 1, Supplementary Digital Content 1, http://links.lww.com/CTG/B384). However, AIH codes were shown to have a low positive predictive value (PPV: 77%) when used alone (27), and required the exclusion of PBC and primary sclerosing cholangitis (PSC) from the records of patients with *ICD-9-CM* and *ICD-10-CM* codes for AIH to increase their PPV for the identification of AIH to 90% (27). This algorithm was validated in previous large database studies (28–30). Therefore, we excluded patients with concurrent PBC and PSC from our study population using *ICD-10-CM* codes (PBC: K74.3; K74.4; K74.5; PSC: K83.0; inflammatory bowel disease [K50/K51] combined with either portal hypertension or esophageal varices [I85/K76.6]). Furthermore, we excluded patients with other CLDs (Figure 1), except MASLD and HCC.

As per the Delphi definition for MASLD, MASLD was defined as the presence of hepatic steatosis (*ICD-10-CM*: K76.0, K75.81), along with the presence of at least 1 metabolic risk factor—overweight or obesity (body mass index >25 kg/m^2^), hypertension (systolic blood pressure ≥130 mm Hg and/or diastolic blood pressure ≥85 mm Hg or current antihypertensive treatment), impaired fasting glucose (≥100 mg/dL, or a prior diagnosis and/or treatment for type 2 diabetes mellitus), hypertriglyceridemia (≥150 mg/dL or treated for high triglycerides), and reduced high-density lipoprotein cholesterol (<40 mg/dL) (Supplementary Table 1, Supplementary Digital Content 1, http://links.lww.com/CTG/B384) (31). The AIH-MASLD cohort included patients with AIH who also met the diagnostic criteria for MASLD. Last, controls were defined as patients with AIH who lacked the diagnostic criteria for MASLD.

### Patient and hospital characteristics

We retrieved data within the TriNetX database on age (mean with SD), sex (male, female), race (White, Black or African American, Asian, American Indian or Alaska Native, Native Hawaiian or Pacific Islander, other races, and unknown races), comorbidities (e.g., celiac disease), and AIH treatment (glucocorticoids (e.g., prednisone, budesonide), azathioprine, 6-mercaptopurine, mycophenolate mofetil, tacrolimus, sirolimus, cyclosporine) and social determinants of adverse health outcomes (SDHOs; defined by the “Z codes” (Z55-Z65), which are endorsed by the American Hospital Association Coding Clinic) (Table 1) (32,33). Using one-to-one (1:1) PSM based on these variables, we matched AIH-MASLD patients to controls (Table 1).

**Table 1.** Baseline characteristics when comparing patients with coexisting AIH and MASLD (AIH-MASLD cohort) with those without MASLD (controls)

Variable	Before PSM			After PSM		
	AIH + MASLD (N = 1,480)	Controls (N = 3,318)	SMD	AIH + MASLD (N = 1,440)	Controls (N = 1,440)	SMD
Age, yr, mean ± SD	53.5 ± 16.9	47.2 ± 21.7	0.323	53.4 ± 16.9	54.1 ± 19.0	0.036
Sex, % (n)						
Female	72.0 (1,066)	73.7 (2,446)	0.038	72.2 (1,040)	69.9 (1,006)	0.052
Male	21.7 (321)	20.9 (692)	0.020	21.7 (313)	23.3 (336)	0.038
Unknown sex	6.3 (93)	5.4 (180)	0.037	6.0 (87)	6.8 (98)	0.031
Race, % (n)						
White	63.3 (937)	61.5 (2,041)	0.037	63.8 (919)	65.0 (936)	0.025
Black or African American	9.8 (145)	15.3 (506)	0.165	10.1 (145)	9.5 (137)	0.019
Asian	6.5 (96)	3.4 (114)	0.141	6.3 (91)	5.8 (84)	0.020
American Indian or Alaska Native	0.8 (12)	0.3 (10)	0.069	0.7 (10)	0.7 (10)	<0.001
Native Hawaiian or Pacific Islander	1.0 (15)	0.5 (18)	0.054	1.0 (14)	0.8 (11)	0.022
Other race	6.4 (94)	5.5 (184)	0.034	6.0 (87)	5.9 (85)	0.006
Unknown race	12.2 (181)	13.4 (445)	0.035	12.2 (175)	12.4 (179)	0.008
Celiac disease, % (n)	2.9 (43)	1.3 (43)	0.112	2.4 (35)	2.6 (38)	0.013
SDHO, % (n)	4.4 (65)	2.5 (84)	0.102	3.9 (56)	3.1 (44)	0.046
Medications, % (n)						
Prednisone	49.5 (732)	44.7 (1,482)	0.096	48.8 (703)	46.7 (673)	0.042
Budesonide	16.3 (241)	10.3 (342)	0.177	15.6 (225)	15.6 (224)	0.002
Azathioprine	29.7 (439)	20.2 (671)	0.219	28.7 (413)	27.4 (395)	0.028
Mercaptopurine	2.5 (37)	1.6 (52)	0.066	2.4 (34)	2.0 (29)	0.024
Mycophenolate mofetil	8.0 (119)	8.5 (281)	0.016	7.8 (112)	6.4 (92)	0.054
Tacrolimus	4.4 (65)	5.9 (197)	0.070	4.3 (62)	3.0 (43)	0.070
Sirolimus	0.7 (10)	0.6 (21)	0.005	0.7 (10)	0.7 (10)	<0.001
Cyclosporine	3.0 (44)	1.8 (59)	0.079	2.7 (39)	2.2 (31)	0.036

Propensity score matching resulted in 1,440 matched pairs. Matching was performed based on demographics (age, sex, race/ethnicity), comorbid conditions (celiac disease), and treatment (prednisone, budesonide, azathioprine, 6-mercaptopurine, mycophenolate mofetil, tacrolimus, sirolimus, cyclosporine).

AIH, autoimmune hepatitis; MASLD, metabolic dysfunction-associated liver disease; SDHO, social determinants of adverse health outcome; SMD, standard mean difference.

### Study aims and outcomes

**Figure 2.**
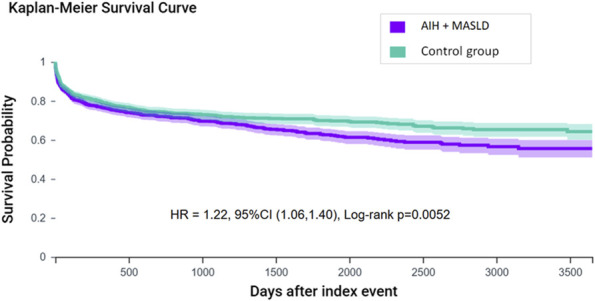
Ten-year progression to cirrhosis in AIH by study group.

Our primary outcome was the development of liver cirrhosis at 10-year follow-up (Figure 2). Multiple secondary outcomes were examined and were divided into short-term (within 1 year of AIH diagnosis) and long-term outcomes (within 10 years of AIH diagnosis). Outcomes were identified using International Classification of Diseases, Tenth Revision; *ICD-10-CM*/*ICD-10-PCS* codes; CPT codes; and TriNetX codes (Supplementary Table 1, Supplementary Digital Content 1, http://links.lww.com/CTG/B384). Short-term outcomes included the rates of 1-year all-cause mortality (TriNetX: Deceased; *ICD-10*: R99), acute liver failure (*ICD-10*: K72.0), liver cirrhosis, immunosuppressive drug use (e.g., prednisone, budesonide, azathioprine, mercaptopurine, mycophenolate mofetil, tacrolimus, sirolimus, cyclosporine, identified with TriNetX codes), liver transplantation (LT) (*ICD-10-CM*: Z94.4, Z48.23; *ICD-10-PCS*: 0FY00Z0, 0FY00Z1, 0FY00Z2; CPT: 47133, 47135, 47140), and all-cause hospitalizations, including critical care unit admissions (CPT: 1013659, 1013729). Long-term outcomes included the rates of 10-year all-cause mortality, liver cirrhosis, hepatocellular carcinoma (HCC) (*ICD-10*: C22.0), immunosuppressive drug use, LT, and all-cause hospitalizations. Liver cirrhosis was defined using a composite of codes for liver cirrhosis (*ICD-10*: K74.6), portal hypertension (*ICD-10*: K76.6), ascites (*ICD-10*: R18) or paracentesis (CPT: 49082, 49083, *ICD-10-PCS*: 0W9G3ZZ, 0W9G30Z), esophageal or gastric varices (*ICD-10*: I85, I86.4) or endoscopic treatment of variceal bleeding (CPT: 43205, 43243, 43244, 43245; *ICD-10-PCS*: 0W3P8ZZ, 0W3P7ZZ), spontaneous bacterial peritonitis (*ICD-10*: K76.7), hepatic encephalopathy (*ICD-10*: K76.82, K72.11, K72.91), hepatorenal syndrome (*ICD-10*: K76.7), and hepatopulmonary syndrome (*ICD-10*: K76.81). Adjustments for confounders were made to account for potential confounders (demographics, SDHOs, comorbid conditions, and treatment). The reporting of this study adheres to the Strengthening the Reporting of Observational Studies in Epidemiology reporting guidelines, provided in the Supplementary Table 2 (see Supplementary Digital Content 1, http://links.lww.com/CTG/B384) (34).

### Statistical analysis

Statistical analyses were conducted using the TriNetX Advanced Analytics Platform. Descriptive statistics for each group are presented as mean ± SD for continuous variables or frequency with proportion for categorical variables. To control for potential confounding variables, 1:1 PSM was performed for comparison between groups (e.g., AIH-MASLD cohort vs controls). Propensity scores were generated, and patients were matched using a greedy nearest-neighbor algorithm with a caliper width of 0.1 SDs. Comparative analyses were conducted within matched groups. For the 10-year risk of liver cirrhosis, Kaplan-Meier analysis was used and comparison was performed using the log-rank test. Hazard ratios (HRs) with 95% confidence intervals (CIs) were estimated using Cox proportional hazards regression among matched samples for all outcomes. Incidence rates were reported as percentages alongside absolute event counts. Baseline characteristics were compared using *t* tests for continuous variables and χ^2^ tests for categorical variables.

## RESULTS

### Study population characteristics

A total of 126,416,530 records were found within the US Collaborative Network database. Among these, 14,225 records for adult patients (≥18 years) who underwent a liver biopsy and were subsequently diagnosed with AIH were identified. After the exclusion of individuals with concurrent PBC, PSC, and other CLDs (e.g., viral hepatitis, alcohol-associated liver disease, toxic liver injury, hemochromatosis, Wilson disease, Budd Chiari syndrome, liver malignancies other than HCC, and biliary obstruction), a final cohort of 4,798 records was included. Within this cohort, 1,480 (31%) met the criteria for MASLD (AIH-MASLD cohort), whereas 3,318 constituted controls (69%) (Figure 1). Owing to insufficient similarity in baseline characteristics, 40 records from the AIH-MASLD cohort and 1,878 controls were excluded. Following 1:1 PSM, 2 balanced cohorts were generated: 2,880 matched records evenly distributed between patients in the AIH-MASLD cohort (n = 1,440) and controls (n = 1,440) (Figure 1). Among unmatched samples, AIH-MASLD patients (n = 1,480) were older (mean age, AIH-MASLD: 53.5 ± 16.9, controls: 47.2 ± 21.7, standard mean difference: 0.036) and had a higher prevalence of White or Asian race, SDHOs, and immunosuppressive therapy compared with controls (n = 3,318) (Table 1). PSM was used, ensuring a balanced distribution of covariates (e.g., all standard mean differences <0.1) (Table 1).

### Short-term outcomes

Short-term outcomes (e.g., within 1 year) among patients with AIH stratified by the presence or absence of MASLD are detailed in Table 2. The AIH-MASLD cohort (n = 1,440) demonstrated significantly reduced 1-year risks of mortality (2.8% vs 4.2%, HR 0.66, 95% CI 0.44–0.98), acute liver failure (1.2% vs 2.2%, HR 0.54, 95% CI 0.30–0.97), and immunosuppressive drug use (57.2% vs 67.8%, HR 0.69, 95% CI 0.63–0.76) compared with controls (n = 1,440). However, no statistically significant differences were observed in the incidence of liver cirrhosis, LT, and all-cause hospitalization rates at 1-year follow-up (Table 2).

**Table 2.** Short-term and mid-to-long-term outcomes when comparing patients with coexisting AIH and MASLD (AIH-MASLD cohort, n = 1,440) with those without MASLD (controls, n = 1,440)

Timeframe	Outcomes	AIH + MASLD % (N)	AIH - MASLD % (N)	HR (95% CI)^a^
Early (within 1 yr)	Short-term mortality	2.8 (41)	4.2 (60)	0.66 (0.44–0.98)
	Acute liver failure	1.2 (17)	2.2 (31)	0.54 (0.30–0.97)
	Liver cirrhosis	22.6 (326)	19.7 (284)	1.14 (0.97–1.34)
	Immunosuppressant use	57.2 (824)	67.8 (977)	0.69 (0.63–0.76)
	Liver transplantation	1.7 (24)	2.0 (29)	0.81 (0.47–1.39)
	All-cause hospitalization	12.4 (179)	12.8 (185)	0.94 (0.77–1.15)
Mid-to-late (within 10 yr)	Long-term mortality	6.4 (92)	8.3 (119)	0.78 (0.60–1.03)
	Liver cirrhosis	30.8 (444)	25.6 (369)	1.22 (1.06–1.40)
	Hepatocellular carcinoma	2.1 (30)	1.0 (15)	2.03 (1.09–3.78)
	Immunosuppressant use	65.3 (940)	74.8 (1,077)	0.70 (0.64–0.77)
	Liver transplantation	2.4 (35)	2.6 (38)	0.91 (0.57–1.43)
	All-cause hospitalization	20.8 (299)	19.7 (283)	1.07 (0.91–1.26)

Patients with preinclusion outcomes were excluded from the analysis.

AIH, autoimmune hepatitis; HR, hazard ratio; MASLD, metabolic dysfunction-associated liver disease.

^a^After propensity score matching (1:1) for demographics, comorbid conditions, and treatment.

### Long-term outcomes

Long-term outcomes (e.g., within a 10-year period) among patients with AIH stratified by the presence or absence of MASLD are detailed in Table 2 and Figure 2. The coexistence of MASLD and AIH (n = 1,440) was associated with significantly higher 10-year risks of liver cirrhosis (30.8% vs 25.6%, HR 1.22, 95% CI 1.06–1.40) and HCC rates (2.1% vs 1.0%, HR 2.03, 95% CI 1.09–3.78). Furthermore, AIH-MASLD patients were less likely to be treated with immunosuppressive medications (65.3% vs 74.8%, HR 0.70, 95% CI 0.64–0.77). However, no statistically significant differences were observed in the 10-year all-cause mortality, LT, and all-cause hospitalizations (Table 2).

## DISCUSSION

In this large cohort study, we investigated the influence of MASLD on the clinical outcomes of individuals with AIH. Patients in the AIH-MASLD cohort showed significantly lower 1-year risks of mortality, acute liver failure, and immunosuppressant use compared with controls, with no significant differences in cirrhosis, LT, and all-cause hospitalizations. Over a 10-year follow-up, the AIH-MASLD cohort exhibited increased risks of cirrhosis and HCC, along with reduced to immunosuppressive exposure; however, long-term all-cause mortality, LT, and hospitalization rates did not differ between groups.

Our findings are consistent with the existing literature, suggesting that AIH-MASLD overlap leads to more severe disease course, with increased liver fibrosis, and higher risks of cirrhosis, HCC, and LT (15,16,18) and often present with more advanced fibrosis and cirrhosis at diagnosis (15,16). This may be due to synergistic inflammatory and fibrotic pathways driven by both autoimmune and metabolic injury. In addition, the presence of metabolic components further exacerbates AIH progression, leading to higher rates of hepatic decompensations, liver-related death, and HCC (16,18). Furthermore, Medina-Morales et al (35) demonstrated that AIH-MASLD patients have higher LT rates and lower waitlist removal risk compared with those with MASLD alone, indicating a more severe disease course. Last, Liu et al (36) found that hepatic steatosis in patients with AIH is associated with poor treatment response and increased fibrosis progression, further supporting the notion that metabolic dysfunction exacerbates AIH progression. Our study is the largest to date including a comprehensive assessment of both short-term and long-term outcomes in patients with AIH-MASLD compared with controls.

Unlike AIH, MASLD is strongly correlated with HCC development because of metabolic comorbidities that induce dysregulation in molecular pathways, including insulin resistance, oxidative stress, and chronic inflammation, which promote progression to cirrhosis and eventually HCC with MASH playing a critical role in escalating chronic inflammation, hepatic stellate cell activation, and fibrosis (13,37,38). Interestingly, while patients in the AIH-MASLD cohort had higher risks of HCC compared with controls, they experienced no significant different in the long-term mortality. Furthermore, patients in the AIH-MASLD cohort remain at increased risk of cardiovascular mortality, although we found no significant differences in the 10-year all-cause mortality between groups.

These findings underscore the need for a personalized approach in managing patients with coexisting AIH and MASLD, especially given the lack of specific guidelines for AIH-MASLD overlap. However, given the increased risk of short-term and long-term liver-related complications, routine liver fibrosis assessment and HCC surveillance should be prioritized. Furthermore, early implementation of lifestyle interventions, including weight reduction and optimization of metabolic and cardiovascular comorbidities (e.g., diabetes, dyslipidemia, and hypertension), is essential to reduce hepatic steatosis, inflammation, and overall cardiovascular morbidity and mortality (13,39). Corticosteroid use in patients with metabolic syndrome warrants caution because of its potential to aggravate metabolic dysfunction. In AIH-MASLD patients, steroid-sparing regimens such as mycophenolate mofetil may be preferable to minimize the risk of worsening insulin resistance and hepatic lipid accumulation. In our cohort, immunosuppressive medication use (including steroids) was lower in patients with AIH-MASLD overlap compared with controls. That, along with the increased 10-year risk of liver cirrhosis warrants further investigations.

The strengths of our study include the use of a large data set of 126,416,530 records, including 14,225 diagnosed with AIH after liver biopsy, enhancing the external validity of our findings relative to smaller studies. PSM adjusted for demographics, comorbidities, and AIH treatment history to attribute observed differences to MASLD. This study has several limitations that should be acknowledged. First, the retrospective design precludes establishing causality. Furthermore, the reliance on ICD codes to identify patients with AIH introduces potential selection bias. However, the exclusion of PBC and PSC cases from the main cohort increases their PPV for the identification of AIH to 90% (27). A key limitation of this study is the absence of liver biopsy results data within the TriNetX platform, which precludes confirmation of AIH based on histologic criteria defined by the International Autoimmune Hepatitis Group (IAIHG) diagnostic framework; this is compounded by the limited availability of autoantibodies and serum immunoglobulin G levels, further restricting the application of diagnostic scoring systems. The reliance on administrative data, specifically *ICD-10* codes, which may introduce misclassification bias, particularly in the context of overlapping CLDs. Although we attempted to mitigate this bias by excluding other CLDs (PBC, PSC, viral hepatitis, alcohol associated liver disease, and others) based on their corresponding *ICD-10* codes, residual misclassification might persist because of variations in coding accuracies across institutions. This may have influenced the observed associations and should be considered when interpreting the results. Last, the absence of liver disease severity markers, such as MELD 3.0 scores or fibrosis staging, restricts a more detailed understanding of the effects of MASLD in patients with AIH.

In summary, our study using real-world evidence showed a significant association between MASLD and worse clinical outcomes in patients with AIH. Future efforts should be targeted toward the implementation of specific guidelines in this patient population to facilitate early detection and management of MASLD in patients with AIH. Larger, prospective studies are necessary to validate these findings and inform evidence-based strategies.

## CONFLICTS OF INTEREST

**Guarantor of the article:** Wing-Kin Syn, MD, PhD.

**Specific author contributions:** Y.K.: collection, analysis, and interpretation of the data and writing of the report. M.A.: analysis and interpretation of the data, and writing of the report. K.Y.T.S.: collection, analysis, and interpretation of the data. M.Y.M.: study supervision, revising the manuscript for important intellectual content, drafting the manuscript. W.-K.S.: study supervision, revising the manuscript for important intellectual content, drafting the manuscript. All authors have approved the final draft submitted.

**Financial support:** None to report.

**Potential competing interests:** None to report.

**Study Highlights**

**WHAT IS KNOWN**

✓ Metabolic dysfunction-associated steatotic liver disease (MASLD) is the most prevalent chronic liver disease worldwide and may complicate the management and outcomes of autoimmune hepatitis (AIH).
✓ Prior studies on AIH-MASLD overlap are limited by small sample sizes.
✓ Immunosuppressive therapy, the cornerstone of AIH management, may worsen MASLD features such as insulin resistance and hepatic steatosis.

**WHAT IS NEW HERE**

✓ This is the largest real-world study to date assessing the impact of MASLD on AIH outcomes, analyzing 2,880 propensity-matched patient records.
✓ AIH-MASLD patients were less likely to receive immunosuppressive treatment compared to controls.
✓ Over 10 years, AIH-MASLD patients demonstrated higher risks of cirrhosis and hepatocellular carcinoma (HCC), despite no differences in long-term mortality and liver transplantation.

## Supplementary Material

## ABBREVIATIONS:

AHA: American Hospital Association
AIH: autoimmune hepatitis
ALF: acute liver failure
ANA: antinuclear antibodies
BMI: body mass index
BP: blood pressure
CI: confidence interval
CLD: chronic liver disease
CPT: current procedural terminology
EBV: epstein–barr virus
EHR: electronic health record
HCC: hepatocellular carcinoma
HCO: health care organization
HDL: high-density lipoprotein
HIV: human immunodeficiency virus
HR: hazard ratio
HRS: hepatorenal syndrome
IAIHG: international autoimmune hepatitis group
ICD: international classification of diseases
IgG: immunoglobulin G
IRB: institutional review board
IS: immunosuppressant
LT: liver transplantation
MASH: metabolic dysfunction-associated steatohepatitis
MASLD: metabolic dysfunction-associated steatotic liver disease
MELD: model for end-stage liver disease
NAFLD: non-alcoholic fatty liver disease
NASH: non-alcoholic steatohepatitis
PBC: primary biliary cirrhosis
PCS: procedure coding system
PPV: positive predictive value
PSC: primary sclerosing cholangitis
PSM: propensity score matching
SBP: spontaneous bacterial peritonitis
SD: standard deviation
SDHO: social determinant of health outcome
SMA: smooth muscle antibodies
SMD: standardized mean difference
STROBE: Strengthening the Reporting of Observational Studies in Epidemiology
