# Supplementary material for: The Impact of Metabolic Dysfunction-Associated Steatotic Liver Disease on Autoimmune Hepatitis Outcomes: A Nationwide Analysis of 2,880 Records
Source: Clin Transl Gastroenterol. 2025 Sep 12;16(11):e00912. doi: 10.14309/ctg.0000000000000912 (PMC12637352; doi:10.14309/ctg.0000000000000912)
Supplement: Supplementary file 1 [file ct9-16-e00912-s001.docx]

**SUPPLEMENTARY MATERIALS**

**Supplementary Table 1.** International Classification of Diseases, Tenth Revision, Clinical Modification / Procedure Coding System (ICD-10-CM/ -PCS) codes, Current Procedural Terminology (CPT)/Healthcare Common Procedure Coding System (HCPCS) Codes, and TriNetX codes for the outcomes in our study population

|  | **Codes and nomenclature** |
| --- | --- |
| **INCLUSION CRITERIA** |  |
| **Autoimmune hepatitis** | ICD-10-CM: K75.4 |
| **Liver biopsy** | CPT:  47000 - Biopsy of liver, needle; percutaneous  47100 - Biopsy of liver, wedge  47001 - Biopsy of liver, needle; when done for indicated purpose at time of other major procedure (List separately in addition to code for primary procedure)  ICD-10-PCS:  0FB00ZX - Excision of Liver, Open Approach, Diagnostic  0FB03ZX - Excision of Liver, Percutaneous Approach, Diagnostic  0FB04ZX - Excision of Liver, Percutaneous Endoscopic Approach, Diagnostic  0FB20ZX - Excision of Left Lobe Liver, Open Approach, Diagnostic  0FB23ZX - Excision of Left Lobe Liver, Percutaneous Approach, Diagnostic  0FB10ZX - Excision of Right Lobe Liver, Open Approach, Diagnostic  0FB13ZX - Excision of Right Lobe Liver, Percutaneous Approach, Diagnostic  0FB24ZX - Excision of Left Lobe Liver, Percutaneous Endoscopic Approach, Diagnostic  0FB14ZX - Excision of Right Lobe Liver, Percutaneous Endoscopic Approach, Diagnostic |
| **EXCLUSION CRITERIA** |  |
| **Primary biliary cirrhosis** | ICD-10-CM: K74.3; K74.4; K74.5 |
| **Primary sclerosing cholangitis** | ICD-10-CM: K83.0 ; (K50/K51) and (I85/K76.6) |
| **Alcohol-associated liver disease** | ICD-10-CM: K70 |
| **Viral hepatitis** | ICD-10-CM: B15-B19 |
| **Wilson's disease** | ICD-10-CM: E83.01 |
| **Hemochromatosis** | ICD-10-CM: E83.11 |
| **Portal or hepatic vein thrombosis** | ICD-10-CM: I81, I82.0 |
| **Warfarin** | TriNetX: RXNORM:11289 |
| **Obstruction of bile duct** | ICD-10-CM: K83.1 |
| **Liver malignancy** (other than hepatocellular carcinoma) | ICD-10-CM:  C22.1 - Intrahepatic bile duct carcinoma  C22.2 - Hepatoblastoma  C22.3 - Angiosarcoma of liver  C22.4 - Other sarcomas of liver  C22.7 - Other specified carcinomas of liver  C22.8 - Malignant neoplasm of liver, primary, unspecified as to type  C22.9 - Malignant neoplasm of liver, not specified as primary or secondary |
| **Metabolic Dysfunction-Associated Steatotic Liver Disease (MASLD)** | Defined as the presence of hepatic steatosis, along with the presence of least one metabolic risk factor - overweight or obesity, hypertension, impaired fasting glucose, hypertriglyceridemia, and reduced high-density lipoprotein (HDL) cholesterol |
| **Liver steatosis** | ICD-10-CM:  K76.0 - Fatty (change of) liver, not elsewhere classified  K75.81 - Nonalcoholic steatohepatitis (NASH) |
| **Overweight and obesity** (e.g. body mass index ≥ 25 kg/m^2^) | TriNetX: TNX:9083 |
| **Hypertension** (e.g. blood pressure ≥ 130/85 mmHg or treated with antihypertensives) | ICD-10-CM:  I10 - Essential (primary) hypertension  TriNetX:  TNX:9085 - systolic BP  TNX:9086 - diastolic BP  NLM:ATC:C09 - agents acting on the renin angiotensin system  NLM:ATC:C03A - thiazide diuretics  NLM:ATC:C08C - calcium channel blockers (vascular)  NLM:ATC:C02 - other antihypertensives |
| **Hypertriglyceridemia** (e.g. serum triglyceride ≥ 150 mg/dL or current treatment for hypertriglyceridemia) | ICD-10-CM:  E78.1 - Pure hyperglyceridemia  TriNetX:  TNX:9004 – serum triglycerides  NLM:ATC: C10AA - statins  NLM:ATC: C10AB - fibrates  RXNORM : 7393 - niacin  RXNORM : 4301 - omega-3 fatty acids |
| **Reduced High-density lipoprotein (HDL) cholesterol** reduced (e.g. HDL < 40 mg/dL) | TriNetX: TNX:9001 |
| **Impaired fasting glucose** (e.g. fasting serum glucose ≥ 100 mg/dL or current treatment for diabetes mellitus) | ICD-10-CM:  E11 - Type 2 diabetes mellitus  TriNetX:  LNC:1558-6 - fasting serum glucose ≥ 100 mg/dL  ATC:A10 - drugs used in diabetes |
| **COMORBID CONDITIONS** |  |
| **Celiac disease** | K90.0 |
| **Social determinants of adverse health outcomes (SDHOs)** | Z55-Z65 |
| **TREATMENT** |  |
| **Prednisone** | TriNetX: RXNORM:8640 |
| **Budesonide** | TriNetX: RXNORM:19831 |
| **Azathioprine** | TriNetX: RXNORM:1256 |
| **6-Mercaptopurine** | TriNetX: RXNORM:103 |
| **Mycophenolate Mofetil** | TriNetX: RXNORM:68149 |
| **Tacrolimus** | TriNetX: RXNORM:42316 |
| **Sirolimus** | TriNetX: RXNORM:35302 |
| **Ciclosporine** | TriNetX: RXNORM:3008 |
| **OUTCOMES** |  |
| **All-cause mortality** | TriNetX: Deceased |
| **Acute liver failure** | ICD-10-CM: K72.0 - Acute and subacute hepatic failure |
| **Liver cirrhosis** | ICD-10-CM:  K74.6 - Other and unspecified cirrhosis of liver  K76.6 - Portal hypertension  R18 - Ascites  I85 - Esophageal varices  I86.4 - Gastric varices  K65.2 - Spontaneous bacterial peritonitis  K76.7 - Hepatorenal syndrome  K76.81 - Hepatopulmonary syndrome  K76.82 - Hepatic encephalopathy  K72.91 - Hepatic failure, unspecified with coma  K72.11 - Chronic hepatic failure with coma  ICD-10-PCS :  0W3P8ZZ - Control Bleeding in Gastrointestinal Tract, Via Natural or Artificial Opening Endoscopic  0W3P7ZZ - Control Bleeding in Gastrointestinal Tract, Via Natural or Artificial Opening  0W9G3ZZ - Drainage of Peritoneal Cavity, Percutaneous Approach  0W9G30Z - Drainage of Peritoneal Cavity with Drainage Device, Percutaneous Approach  CPT :  43205 - Esophagoscopy, flexible, transoral; with band ligation of esophageal varices  43243 - Esophagogastroduodenoscopy, flexible, transoral; with injection sclerosis of esophageal/gastric varices  43244 - Esophagogastroduodenoscopy, flexible, transoral; with band ligation of esophageal/gastric varices  43245 - Esophagogastroduodenoscopy, flexible, transoral; with dilation of gastric/duodenal stricture(s) (eg, balloon, bougie)  49082 Abdominal paracentesis (diagnostic or therapeutic); without imaging guidance  49083 Abdominal paracentesis (diagnostic or therapeutic); with imaging guidance |
| **Hepatocellular carcinoma** | ICD-10-CM: C22.0 |
| **Liver transplantation** | CPT:  47133 - Donor hepatectomy (including cold preservation), from cadaver donor  47135 - Liver allotransplantation, orthotopic, partial or whole, from cadaver or living donor, any age  47140 - Donor hepatectomy (including cold preservation), from living donor; left lateral segment only (segments II and III)  ICD-10-PCS:  0FY00Z0 - Transplantation of Liver, Allogeneic, Open Approach  0FY00Z1 - Transplantation of Liver, Syngeneic, Open Approach  0FY00Z2 - Transplantation of Liver, Zooplastic, Open Approach  ICD-10-CM:  Z94.4 - Liver transplant status  Z48.23 - Encounter for aftercare following liver transplant |
| **All-cause hospitalizations** (including critical care unit admissions) | CPT: 1013659, 1013729 |

**Supplementary Table 2**. STROBE Statement

|  | Item No | Recommendation | Page No |
| --- | --- | --- | --- |
| **Title and abstract** | 1 | (*a*) Indicate the study’s design with a commonly used term in the title or the abstract | 1,2 |
|  |  | (*b*) Provide in the abstract an informative and balanced summary of what was done and what was found | 2 |
| Introduction | | | |
| Background/rationale | 2 | Explain the scientific background and rationale for the investigation being reported | 3 |
| Objectives | 3 | State specific objectives, including any prespecified hypotheses | 4-5 |
| Methods | | | |
| Study design | 4 | Present key elements of study design early in the paper | 6 |
| Setting | 5 | Describe the setting, locations, and relevant dates, including periods of recruitment, exposure, follow-up, and data collection | 6 |
| Participants | 6 | (*a*) Give the eligibility criteria, and the sources and methods of selection of participants. Describe methods of follow-up | 6 |
|  |  | (*b*) For matched studies, give matching criteria and number of exposed and unexposed | 8-9 |
| Variables | 7 | Clearly define all outcomes, exposures, predictors, potential confounders, and effect modifiers. Give diagnostic criteria, if applicable | 6-8 |
| Data sources/ measurement | 8* | For each variable of interest, give sources of data and details of methods of assessment (measurement). Describe comparability of assessment methods if there is more than one group | 5, 9 |
| Bias | 9 | Describe any efforts to address potential sources of bias | 9 |
| Study size | 10 | Explain how the study size was arrived at | 5, 6 |
| Quantitative variables | 11 | Explain how quantitative variables were handled in the analyses. If applicable, describe which groupings were chosen and why | 9 |
| Statistical methods | 12 | (*a*) Describe all statistical methods, including those used to control for confounding | 9 |
|  |  | (*b*) Describe any methods used to examine subgroups and interactions |  |
|  |  | (*c*) Explain how missing data were addressed |  |
|  |  | (*d*) If applicable, explain how loss to follow-up was addressed |  |
|  |  | (*e*) Describe any sensitivity analyses |  |
| Results | | |  |
| Participants | 13* | (a) Report numbers of individuals at each stage of study—eg numbers potentially eligible, examined for eligibility, confirmed eligible, included in the study, completing follow-up, and analysed | 10 |
|  |  | (b) Give reasons for non-participation at each stage |  |
|  |  | (c) Consider use of a flow diagram |  |
| Descriptive data | 14* | (a) Give characteristics of study participants (eg demographic, clinical, social) and information on exposures and potential confounders | 10 |
|  |  | (b) Indicate number of participants with missing data for each variable of interest |  |
|  |  | (c) Summarise follow-up time (eg, average and total amount) |  |
| Outcome data | 15* | Report numbers of outcome events or summary measures over time | 11 |

| Main results | 16 | (*a*) Give unadjusted estimates and, if applicable, confounder-adjusted estimates and their precision (eg, 95% confidence interval). Make clear which confounders were adjusted for and why they were included | 11 |
| --- | --- | --- | --- |
|  |  | (*b*) Report category boundaries when continuous variables were categorized |  |
|  |  | (*c*) If relevant, consider translating estimates of relative risk into absolute risk for a meaningful time period |  |
| Other analyses | 17 | Report other analyses done—eg analyses of subgroups and interactions, and sensitivity analyses | 11 |
| Discussion | | | |
| Key results | 18 | Summarize key results with reference to study objectives | 12 |
| Limitations | 19 | Discuss limitations of the study, taking into account sources of potential bias or imprecision. Discuss both direction and magnitude of any potential bias | 14 |
| Interpretation | 20 | Give a cautious overall interpretation of results considering objectives, limitations, multiplicity of analyses, results from similar studies, and other relevant evidence | 12-14 |
| Generalizability | 21 | Discuss the generalizability (external validity) of the study results | 14 |
| Other information | | | |
| Funding | 22 | Give the source of funding and the role of the funders for the present study and, if applicable, for the original study on which the present article is based | N/A |
